# Abdominal wall muscle remodeling after open transversus abdominis release: a CT-based analysis

**DOI:** 10.1007/s10029-026-03630-w

**Published:** 2026-03-11

**Authors:** Jessica Zilberman Macret, Pedro Henrique de Freitas Amaral, Eduardo Rullo Maranhão Dias, João Paulo Venâncio de Carvalho, Giovanna Zucchini Rondini, Sergio Roll

**Affiliations:** Santa Casa de Sao Paulo - Abdominal Wall Surgery Unit, Rua Cesário Mota Júnior, 112 - Vila Buarque, São Paulo, SP Brazil

**Keywords:** Abdominal wall reconstruction, Complex ventral hernia, Transversus abdominis release, Computed tomography, Muscle remodeling

## Abstract

**Purpose:**

To evaluate abdominal wall muscle remodeling after posterior component separation using the transversus abdominis release (TAR) technique, and to explore potential associations between muscle remodeling and clinical, demographic, and anatomical variables.

**Methods:**

This retrospective study included adults with incisional ventral hernia who underwent abdominal wall reconstruction with TAR between 2019 and 2023. Pre- and postoperative abdominal CT scans (≥ 6 months) were analyzed to measure bilateral cross-sectional areas of the rectus abdominis (RA), internal oblique (IO), and external oblique (EO) muscles at L3–L4 level. Percentage variation in muscle cross-sectional area (CSA), calculated using bilateral mean values, was used to characterize morphometric remodeling. Associations with demographic factors, clinical variables, hernia characteristics, and postoperative outcomes were assessed using Wilcoxon and Spearman tests.

**Results:**

Thirty-seven patients met inclusion criteria. Paired analyses demonstrated a consistent postoperative increase in CT-derived muscle CSA across RA, IO, and EO (all *p* < 0.001). In exploratory analyses, no demographic, clinical, or anatomic variable demonstrated a statistically significant association with bilateral mean muscle remodeling.

**Conclusion:**

Patients undergoing open TAR demonstrate consistent postoperative abdominal wall morphometric remodeling on CT, characterized by RA expansion and coordinated changes in the oblique muscles following midline restoration. The clinical significance of these imaging findings remains uncertain and warrants prospective studies integrating functional outcomes.

## Introduction

Ventral incisional hernias are common complications after abdominal operations and frequently lead to progressive structural and functional impairment of the abdominal wall musculature, particularly in large or chronic defects where lateralization contributes to muscle atrophy and loss of core stability [1–3]. Posterior component separation with transversus abdominis release (TAR) has become an established approach for complex abdominal wall reconstruction, enabling reliable midline restoration and improved tension-free closure [4–6].

Radiographic studies have demonstrated that anatomical realignment after TAR may promote compensatory morphological remodeling of the abdominal wall muscles [7]. However, these morphological adaptations have not been consistently correlated with demographic or clinical characteristics, and the factors potentially influencing postoperative muscle remodeling remain poorly understood [7, 8]. Computed tomography (CT) provides an objective method for evaluating muscle morphology and may help clarify the factors influencing this adaptive response.

Thus, the present study aims to quantify CT-based remodeling of the rectus abdominis, internal oblique, and external oblique muscles after TAR, and to explore potential associations between postoperative muscle remodeling and demographic, clinical and anatomical variables.

## Methods

### Ethical approval

The study was approved by the Ethics Committee of the Irmandade da Santa Casa de Misericórdia de São Paulo (CAAE 47422021.0.0000.5479). All participants provided written informed consent, and the study adhered to national regulatory guidelines.

### Data source and patient selection

This was a retrospective analysis of a prospectively maintained database including adult patients with incisional ventral hernia who underwent abdominal wall reconstruction with transversus abdominis release (TAR) between January 2019 and December 2023. Medical records were reviewed to collect demographic variables (age, sex, body mass index- BMI), clinical characteristics (diabetes mellitus- DM, smoking), anatomical data (hernia location and defect width), and postoperative outcomes (complications and recurrence).Pre- and postoperative abdominal computed tomography (CT) scans performed at least six months apart were required for inclusion. Exclusion criteria were incomplete records, loss to follow-up, withdrawal of consent, or preoperative use of botulinum toxin A.

### Surgical technique

All procedures were performed following a standardized open TAR protocol used by the institutional abdominal wall reconstruction team. The technique involved posterior component separation with division of the posterior rectus sheath and controlled release of the transversus abdominis muscle, followed by retromuscular mesh placement and midline restoration using polypropylene mesh.

### CT-based muscle analysis

Bilateral cross-sectional areas (CSA) of the rectus abdominis (RA), internal oblique (IO), and external oblique (EO) were measured on axial CT images at the L3–L4 level, a landmark widely used for quantitative abdominal wall morphometry and previously adopted in radiographic analyses after TAR [7].

**Fig. 1:**
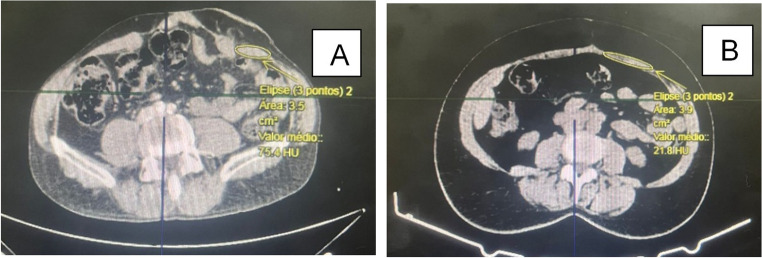
**A**) Preoperative left RA measurement; **B**) Postoperative left RA measurement

To avoid assumptions inherent to volumetric estimations, we did not derive muscle volumes from linear dimensions; instead, we quantified CSA at a standardized anatomical level. All CT measurements were performed using a predefined protocol: (i) identification of the mid–L3/L4 intervertebral disc level, (ii) consistent axial slice selection, and (iii) manual delineation of the muscle borders for RA, IO, and EO bilaterally. When muscle borders were less distinct, adjacent slices were reviewed to confirm fascial planes and to maintain anatomical consistency. Measurements were performed by a single examiner blinded to clinical outcomes, using identical software settings across cases (Fig. 1).

For each muscle group, CSA was measured separately on the right and left sides. To obtain a single patient-level morphometric value and reduce side-specific variability, a bilateral mean CSA was calculated as the arithmetic mean of the right and left measurements at each time point (preoperative and postoperative). Muscle remodeling was then quantified as the percentage variation between pre- and postoperative bilateral mean values. This approach was chosen to represent global abdominal wall remodeling after midline restoration rather than side-specific changes.

### Statistical analysis

Descriptive statistics were used to summarize demographic, clinical, and anatomical variables. Continuous variables were expressed as medians (MED) and interquartile ranges (IQR), and categorical variables as absolute numbers and percentages.

Given the modest sample size and the risk of inflated type I error due to multiple comparisons, primary morphometric endpoints were predefined as the bilateral mean CSA change for each muscle group (RA, EO, IO). By averaging bilateral CSA values prior to percentage change calculation, we aimed to minimize the influence of local asymmetries and focus on overall abdominal wall remodeling as the primary biological endpoint. This predefined endpoint was selected a priori to reflect global abdominal wall adaptation following midline restoration rather than localized or side-dependent changes. Paired comparisons between pre- and postoperative CSA for these primary endpoints were performed using the Wilcoxon signed-rank test due to non-normal distribution.Associations between percentage CSA variation of the primary endpoints and categorical variables (sex, BMI ≤30 or >30, diabetes, smoking, hernia location, postoperative complications, and recurrence) were evaluated using the Wilcoxon rank-sum test. Correlations with continuous variables (age, defect width, and postoperative interval) were assessed using Spearman’s rank correlation coefficient.

Side-specific (right/left) analyses and subanalyses in patients with lateral hernias were considered exploratory, performed to describe potential asymmetries, and were not used to support primary inferential conclusions. Statistical significance was set at p < 0.05 for primary analyses. All analyses were performed using R software.

## Results

**Fig. 2:**
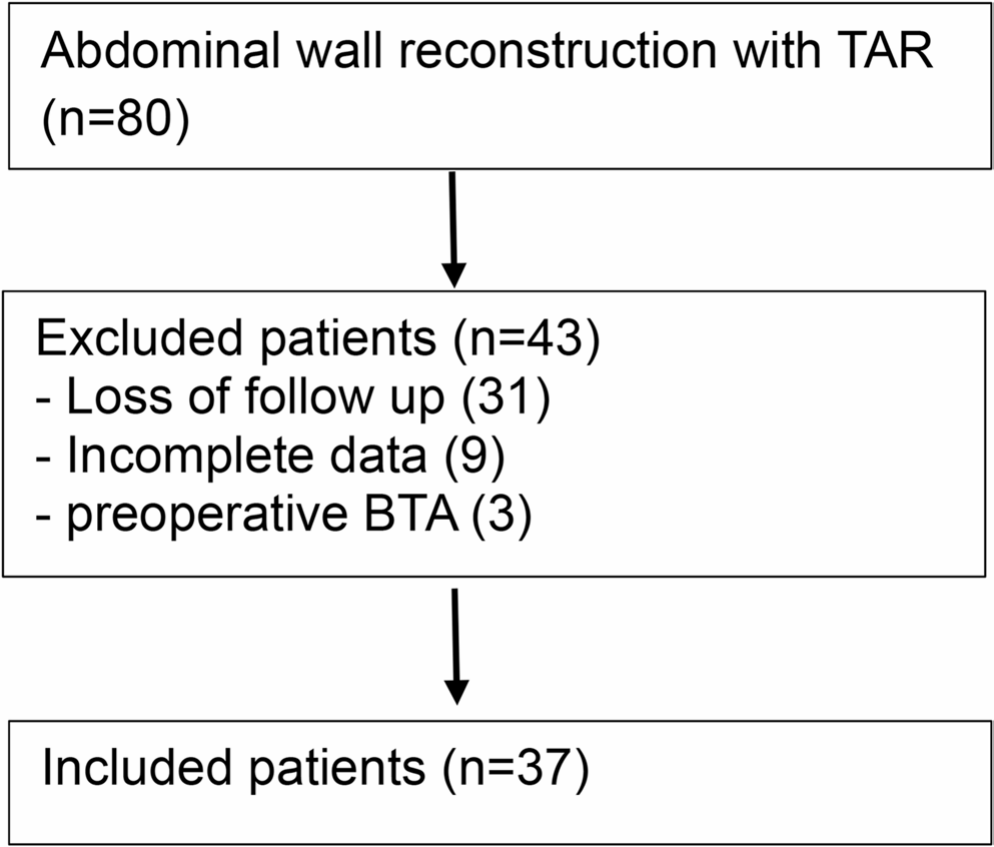
Included and excluded patients

A total of 80 patients underwent TAR during the study period. Of these, 37 met the inclusion criteria and were analyzed; exclusions were due to loss to follow-up, incomplete clinical or imaging data, or preoperative botulinum toxin A (BTA) use (Fig. 2).

### Patient characteristics

The final cohort included 16 women (43.2%) and 21 men (56.8%), with a median age of 57 years (IQR 49–66) and median BMI of 26.9 kg/m² (IQR 24.3- 29.5). Fifteen patients (40.5%) were smokers and six (16.2%) had diabetes mellitus. The median transverse hernia width was 8.4 cm (IQR 6.8- 10), and most defects were located in the midline (n = 23; 62.2%). Postoperative complications occurred in five patients (13.2%), and two (5.4%) developed recurrence during follow-up (Tables 1 and 2).

**Table 1:** Demographic and clinical characteristics of the study cohort (*n* = 37)

Variables	Results
Sex	
Women	16 (43,2%)
Man	21 (56,8%)
Age (years)	57 (49–66)
BMI (kg/m²)	26,9 (24.3–29.5)
Smoking	15 (40,5%)
DM	6 (16,2%)

**Table 2:** Hernia characteristics and postoperative outcomes

Variables	Results
Hernia width (cm) Localization	8,4 (6.8–10)
Midline	23 (62,2%)
Lateral	14 (37,8%)
Postoperative complications	5 (13,2%)
Hernia recurrence	2 (5,4%)

### Overall muscle remodeling

Using predefined primary morphometric endpoints based on bilateral mean muscle cross-sectional area (CSA), paired analyses demonstrated a consistent and significant postoperative increase in all evaluated abdominal wall muscle groups following TAR.The rectus abdominis showed a significant postoperative CSA expansion (median 16.1% [IQR 11.4- 27], p < 0.001). Similar increases were observed in the internal oblique (median 16,5% [IQR 8.5–20.1.5.1], p < 0.001) and external oblique muscles (median 9.5% [IQR 6.5–12.7.5.7], p < 0.001). These findings indicate a uniform morphometric remodeling response across the abdominal wall musculature after midline restoration. Results are summarized in Table 3.

**Table 3:** Primary CT-based morphometric outcomes using bilateral mean muscle CSA

**Muscle**	**Preoperative CSA**	**Postoperative CSA**	△**CSA%**	**p-value**
RA	3.85 [3.16–4.99.16.99]	4.43 [3.95–5.88.95.88]	16.1 [11.4–27.4]	<0.001
IO	5.15 [4.38–5.68.38.68]	6 [4.98–6.86.98.86]	16.5 [8.5–20.1.5.1]	<0.001
EO	4.15 [3.44–5.18.44.18]	4.73 [3.94–5.53.94.53]	9.5 [6.5–12.7.5.7]	<0.001

### Side-specific muscle remodeling (exploratory analysis)

**Fig. 3:**
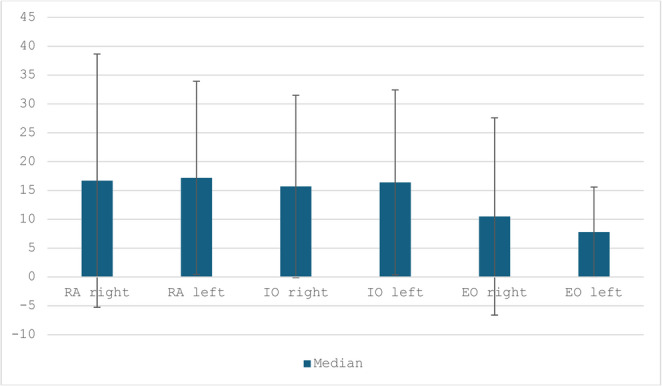
Note: This is mandatory. Please Provide

Exploratory side-specific analyses demonstrated symmetric postoperative CSA expansion of the rectus and oblique muscles. Median percentage CSA increases were comparable between right and left sides for all muscle groups (Fig. 3). These analyses were performed to describe potential asymmetries and were not used to support primary inferential conclusions.

### Associations with clinical and demographic variables

No statistically significant associations were identified between bilateral mean muscle remodeling and any evaluated demographic or clinical variable. Although numerical differences were observed across several subgroups (Table 4), these did not reach statistical significance and should be interpreted as descriptive only.

**Table 4:** Associations between demographic/clinical variables and bilateral mean percentage CSA variation

Variable	Muscle	Group 0	Group 1	*p*-value
Smoking (No vs. Yes)	RA	17.2 [13.8–30.5]	14 [7.4–22.7]	0.095
	IO	17.3 [11.7–19.9]	16 [6.5–20.1]	0.733
	EO	10.2 [7–16.3.3]	8.4 [4–9.8.8]	0.131
Sex (Female vs. Male)	RA	19.1 [14.7–29.9]	14.1 [8.6–20.3]	0.095
	IO	15.7 [4.4–18.6]	18.6 [10.8–27.7]	0.139
	EO	8.9 [4.4–10.8]	9.5 [7.3–17.5]	0.332
BMI (*≤* 30 vs. > 30)	RA	14.9 [9.5–26.1]	17.3 [15–27.6.6]	0.266
	IO	17.3 [7.6–24.7]	16.4 [13.4–18.4]	0.737
	EO	9.2 [5.3–12.9]	10.1 [8.5–12.1]	0.266
DM (No vs. Yes)	RA	16.4 [9–29.5.5]	16.2 [15–17.6.6]	0.881
	IO	16.5 [9–26.3.3]	15.5 [6–18.5.5]	0.454
	EO	9.3 [5–17.3.3]	10 [9.1–10.7]	0.749
Hernia Location (Midline vs. Lateral)	RA	15.7 [10.1–25.6]	16.4 [13.5–28.3]	0.869
	IO	17.2 [10.2–24.2]	16.5 [6.2–19.8]	0.645
	EO	9.2 [5.5–12.8]	9.9 [7.5–12.1]	0.693
Recurrence (No vs. Yes)	RA	16.7 [12.6–27.4]	9 [3.6–15.1]	0.122
	IO	16.5 [10.9–19.9]	11.4 [6.2–31.7]	0.756
	EO	9.9 [7.2–12.8]	6.7 [2.2–9.7]	0.325

Overall, bilateral mean muscle remodeling demonstrated limited variability across patient subgroups, with no clinically meaningful predictors identified in this cohort.

No statistically significant correlations were observed between bilateral mean percentage CSA variation and age, hernia width, or postoperative interval for any muscle group (all p > 0.05). These analyses are reported as exploratory and descriptive (Table 5).

**Table 5:** Spearman correlations between continuous variables and bilateral mean percentage CSA variation

Variable	Muscle	Spearman coefficient	*p*-value
Age (Years)	RA	−0.21	0.22
	IO	0.12	0.5
	EO	−0.11	0.54
Hernia widht (cm)	RA	0.16	0.35
	IO	0.08	0.63
	EO	0.12	0.48
Postoperative interval (months)	RA	0.11	0.53
	IO	−0.03	0.88
	EO	0.22	0.19

### Lateral hernia subanalysis

An exploratory subanalysis was performed in patients with lateral hernias to descriptively compare ipsilateral and contralateral muscle CSA changes. Although a trend toward lower CSA expansion on the side of the defect was observed, no statistically significant differences were identified for any muscle group (all p > 0.05). Given the limited sample size, these findings should be interpreted as descriptive and hypothesis-generating only.

## Discussion

Although postoperative increases in CT-derived muscle CSA were consistently observed, CT morphometry cannot distinguish true myofiber hypertrophy from changes related to muscle reorientation, lengthening, or medialization following midline restoration. Therefore, the present findings should be interpreted as evidence of morphometric remodeling, rather than definitive physiological hypertrophy, and their clinical implications framed accordingly.

In this study, abdominal wall reconstruction using the TAR technique was associated with a uniform and significant postoperative increase in the CSA of all evaluated abdominal wall muscle groups, including the rectus abdominis, internal oblique, and external oblique muscles. These findings suggest that anatomical midline restoration itself may represent the dominant stimulus for postoperative abdominal wall morphometric adaptation.

When predefined primary endpoints based on bilateral mean CSA variation were applied, these changes were consistent across patients and independent of baseline demographic or clinical characteristics. These findings reinforce the concept that midline restoration itself represents a central stimulus for abdominal wall muscular adaptation, even in the setting of transversus abdominis division.

Concerns regarding potential functional impairment after transversus abdominis section have been discussed in the literature, given its essential contribution to trunk stability and tensioning of the thoracolumbar fascia [3, 8]. Nevertheless, prior studies indicate that core function is preserved or even improved after TAR. Haskins et al. reported significant enhancements in core stability, low-back pain, and quality of life after TAR without measurable compromise in muscle performance at six months [8]. Similarly, Criss et al. demonstrated increased abdominal wall strength and improved quality-of-life metrics following midline restoration, emphasizing the interplay between structural correction and functional recovery []. While functional assessment was not performed in the present study, the consistent morphometric response observed supports the biological plausibility of these clinical findings.

Our findings align closely with previous radiographic analyses. De Silva et al. showed substantial hypertrophy of all major muscle groups after TAR, whereas bridging repairs failed to demonstrate similar adaptive changes [7]. This contrast underscores the importance of anatomical realignment anda midline reconstitution—rather than mesh reinforcement alone—in driving muscular remodeling. More recently, Daes et al. evaluated post-eTEP changes in patients with small ventral hernias and diastasis, demonstrating increases in rectus abdominis area and improved abdominal contour, suggesting that anatomical reconstruction may create favorable conditions for subsequent muscle remodeling, particularly when combined with structured postoperative conditioning [10].

Clinically, these findings reinforce that abdominal wall reconstruction not only restores structural integrity but also creates biomechanical conditions that appear to favor compensatory muscle remodeling. This response is likely mediated by improved vector alignment, reestablishment of central fascial tension, and redistribution of mechanical load [3, 7, 9]. While muscle remodeling alone does not necessarily imply functional improvement, emerging evidence indicates that anatomical reconstruction may enable secondary physiological benefits when coupled with postoperative rehabilitation [11, 12]. Further studies integrating CT morphometry with objective functional metrics are needed to clarify this relationship.

Importantly, no demographic, clinical, or anatomical variable demonstrated a statistically significant association with bilateral mean muscle remodeling in the present cohort. While numerical differences were observed across certain subgroups, including smoking status, sex, hernia size, and postoperative interval, these findings did not reach statistical significance and should be interpreted as descriptive and hypothesis-generating only. The absence of strong predictors suggests that the morphometric response to TAR may be relatively robust across diverse patient profiles, further supporting the central role of anatomical restoration in driving postoperative remodeling.

In exploratory analyses, smoking was associated with numerically lower rectus abdominis remodeling; however, this observation did not persist across all primary endpoints and did not achieve statistical significance when bilateral mean measures were applied. Although biologically plausible given the known adverse effects of nicotine on microvascular perfusion, collagen synthesis, and anabolic capacity [13], this finding should be interpreted cautiously and warrants validation in larger, prospectively designed studies. Similarly, older age demonstrated weak, non-significant correlations with muscle remodeling, potentially reflecting age-related alterations in muscle plasticity, including reduced satellite cell activation and increased intramuscular fibrosis [14, 15].

In the exploratory subanalysis of lateral hernias, a non-significant trend toward reduced CSA expansion on the ipsilateral side was observed. Given the limited sample size, this finding is descriptive only and does not support lateralized biological inference. Nevertheless, it highlights the potential heterogeneity of muscular adaptation in the presence of pre-existing unilateral anatomical disruption and underscores the need for larger studies specifically addressing lateral hernia populations.

From a clinical perspective, the present findings support the broader concept of functional abdominal wall reconstruction, in which restoring the linea alba reestablishes muscular orientation and facilitates physiological adaptation. Although direct functional measurements were not included in this study, the demonstrable and consistent morphological remodeling response suggests that midline restoration is a biologically active intervention with implications extending beyond defect closure.

This study has limitations. The sample size was modest, and more than half of the initial cohort was lost to follow-up, reflecting common challenges in complex abdominal wall reconstruction populations, including socioeconomic barriers, transportation limitations, and postoperative attrition [16, 17]. Second, CSA measurements were performed by a single examiner and we did not include inter- or intra-observer reproducibility testing in this dataset, which may introduce measurement variability; however, we minimized this risk through a standardized acquisition and measurement protocol at a fixed anatomical landmark (L3–L4) and by avoiding indirect volumetric estimations. Additionally, morphometric assessment was limited to static CT-based measurements, precluding conclusions regarding muscle strength or functional performance. Finally, the retrospective design limits control over imaging intervals and patient heterogeneity.

In conclusion, open TAR is associated with consistent postoperative abdominal wall morphometric remodeling on CT, characterized by rectus abdominis CSA expansion and coordinated changes in the oblique muscles following midline restoration. While the functional and clinical implications of these imaging findings remain to be fully defined, the consistent morphometric response observed supports the biological relevance of midline restoration beyond defect closure alone and warrants prospective studies integrating morphometry with objective functional and patient-reported outcomes.
